# Hyperglycemia in Severe Falciparum Malaria: A Case Report

**DOI:** 10.1155/2012/312458

**Published:** 2012-10-17

**Authors:** Leonardo Chianura, Isabella Corinna Errante, Giovanna Travi, Roberto Rossotti, Massimo Puoti

**Affiliations:** Department of Infectious Diseases, Niguarda Cà Granda Hospital, Piazza Ospedale Maggiore 3, 20162 Milano, Italy

## Abstract

Occasionally, malaria may present with unusual signs and symptoms. We report a case of an uncommon presentation of *Plasmodium falciparum* infection in a 59-year-old Ethiopian immigrant, which initially presented with hyperglycaemia and multiple organ dysfunction syndrome (MODS). Reports of unusual presentations of malaria are few and cases of severe malaria with hyperglycaemia are rarely described. As hyperglycaemia is associated to most severe malaria and high mortality, our aim is to catch the attention of the physicians on this entity.

## 1. Introduction

Severe imported malaria remains a major threat to travelers. We describe a case of severe P. falciparum imported malaria with hyperglycaemia and MODS. Although rare, this syndrome should be considered in patients brought to the emergency department after travelling to an area where malaria is endemic.

## 2. Case Report

The patient was a 56-year-old Ethiopian immigrant who moved to Italy in 1974. Symptoms started 17 days after returning from a 6-week holiday trip to Cote d'Ivoire (June 28–August 8, 2004). The patient had stayed both in rural and urban areas. She had not taken any malaria chemoprophylaxis during the trip. Three days after onset of fever with recurrent vomiting episodes she referred to the family doctor and she was treated with antibiotic; malaria was not clinically suspected. On arrival on August 25, the patient was dehydrated and pyrexial with a temperature of 37.8°C. Initial laboratory studies showed high creatinine level (2.42 mg/dL; reference range 0.50–1.10), uremia (urea 192 mg/dL; reference range 18–48), hyperglycaemia (glucose 794 mg/dL; reference range 70–110), lactic acidosis (lactic acid 3.1 mmoli/L; reference range 0.60–1.80), hypocalcemia (calcium 6.56 mg/dL; reference range 8.50–10.50), hyponatraemia (sodium 129 mmoli/L; reference range 132–143), anaemia (haemoglobin 7.9 g/dL; reference range 12.0–16.0), thrombocytopenia (platelet count 6 × 10^9^/L; low-normal 140 × 10^9^/L), and high levels of C-reactive protein (CRP) (CRP 13.4 mg/dL; reference range = 0.0–0.5 mg/dL). The results of the rest of the routine blood tests were within normal limits. A blood smear detected a *P. falciparum*, with a density of 40% of parasitized red blood cells (RBCs). A chest X-ray performed showed right middle lobe pneumonia and moderate bilateral pleural effusion. The patient was screened for Human immunodeficiency virus, Parvovirus B19, Hepatitis B virus, Hepatitis C virus, Coxsackie B 1–6, Cytomegalovirus; Herpes Simplex Virus 1 and 2, Epstein-Barr virus, and enteric fever. The patient was admitted to intensive care unit (ICU) and she was treated for 7 days with intravenous quinine 10 mg/kg every 8 hours after an initial loading dose of 20 mg/kg, for 7 days with doxycycline 100 mg.iv. every 12 hours and for 14 days with ceftriaxone 2 gr once a day. Hyperglycemia was controlled by applying an intensive insulin protocol with a target glycemia of 150 mg/dL. During the acute phase the patient was described with angina pectoris with normal troponin T levels; ECG showed T-wave alterations and echocardiography a global left ventricular hypokinesia; a supportive care with dopamine and nitrates was necessary. On followup, ECG and echocardiography had normalized. From August 25 to August 29, 2004 she was transfused with 6 units of compatible packed red cells (PRC), 3 unit random platelet concentrates (RPCs), and 4 units of fresh-frozen plasma (FFP) obtained by apheresis.

24 hours after starting quinine the parasitemia decreased by 50% and less insulin was needed to achieve glucose control. Thick and thin smears obtained 72 hours after therapy were negative for parasites. 

The insulin was injected subcutaneously according to blood glucose control since September 2. She did not need insulin since September 3. Her fasting blood sugar was in the normal range when discharged on September 9, 2004.

## 3. Discussion

Malaria remains a major cause of morbidity and mortality worldwide. An estimated 300–500 million people contract malaria each year, resulting in 1.5–2.7 million deaths annually [1]. 

The classic presentation of malaria consists of paroxysms of fever. Symptoms usually associated with febrile paroxysms include shaking chills, sweats, headache, rigors, fatigue, malaise, arthralgia, myalgia, back pain, abdominal pain, nausea, vomiting, diarrhoea, and jaundice [2]. 

However, classical presentation is seen in only 50%–70% of the cases with the rest having atypical manifestations. 

Some patients can develop or present signs or symptoms that suggest complications of malaria, usually caused by *Plasmodium falciparum*, such as cerebral malaria (CM) defined as coma or altered mental status, cerebellar ataxia or multiple seizures, seizures secondary to either hypoglycemia or cerebral malaria, renal failure, severe anaemia, thrombocytopenia, hypoglycemia, hemoglobinuria (blackwater fever), noncardiogenic pulmonary edema, ARDS, renal failure, lactic acidosis, multiple organ dysfunction, bleeding (coagulopathy), and hemolysis resulting in severe anemia and jaundice.

It is important to be aware that malaria may present with unusual presentations. Uncommon presentations of malaria more frequent in *P. falciparum* malaria are acute abdomen, viral hepatitis-like illness, uremic encephalopathy, Guillain-Barré syndrome, severe headache, focal deficit (hemiplegia), urticaria, subacute intestinal obstruction, hyperglycemia, pancytopenia, pernicious syndrome, urinary frequency, relative bradycardia, and unexplained shock [1–3]. 

Hyperosmolar hyperglycemic state (HSS) most commonly occurs in patients with type 2 diabetes mellitus who have some concomitant illness that leads to reduced fluid intake. In general, any illness that predisposes to dehydration may lead to HHS. Infections are the major precipitating factor with urinary tract infections and pneumonia being the most common underlying causes of HHS [4]. 

Other examples of acute conditions that may lead to HHS are stroke, intracranial hemorrhage, myocardial infarction, pancreatitis, trauma or severe burns, and pulmonary embolism. Stress response to any acute illness tends to provoke the release of counterregulatory hormones such as cortisol, catecholamines, and glucagons that favor elevated glucose levels. 

HSS is a potentially life-threatening emergency. Hospital treatment forHHS involves replacing the lost fluid caused by high glucose levels and the administration of insulin through a vein, to bring the blood glucose down to an acceptable level. It does not usually lead to the presence of ketones in the urine, as what occurs in ketoacidosis. 

Osier et al. reported that hyperglycaemia in the absence of insulin-dependent diabetes mellitus was present in 2.7% of children admitted to a rural Kenyan district hospital; the observational study included 3462 children outside the neonatal period and the commonest main primary diagnose was malaria (49.4%) [5]. Moreover, they observed the mortality in hyperglycaemic children was higher than that in normoglycaemic children. 

Van Thien et al. reported the association of hyperglycaemia with severe malaria (SM) and that CM stimulates glucose production to a greater extent than other forms of malaria [6]. 

Tombe et al. described one case of fatal SM with hyperglycaemia in a series of 33 patients [7]. Dass et al. saw two cases of hyperglycaemia in malaria in a series of 162 cases who responded well to insulin therapy [8]. 

Eltahir et al. reported the hyperglycaemia was frequent in SM but more commonly associated with CM; the bood glucose levels were even higher in the fatal cases of CM [9]. 

On arrival our patient was dehydrated and pyrexial. We believe that malaria and associated pneumonia have led to a range of responses: from increased counter regulatory hormones with hyperglycemia to a systemic inflammatory response (SIRS) which results in MODS. 

Prompt treatment with intravenous (IV) rehydration, IV quinine sulphate, continuous IVs insulin, and ceftriaxone has led to clinical improvement.

Moreover, during the acute phase we observed clinical findings of myocarditis with ECG abnormalities and global left ventricular hypokinesia. Cardiac complications are extremely rare in malaria. Myocarditis has been found in some autopsy studies. However, a few cases of myocarditis have been reported and only during severe or fatal *P. falciparum* malaria and published echocardiographic and electrocardiographic data are rare [10, 11]. 

A relationship between the cardiac event and the parasite challenge seems probable, especially because of the chronology of the event and the apparent absence of underlying viral infections. On followup, the ECG and echocardiography had normalized. As observed by Günther et al. persistent cardiac damage following malarial infection is rare [12]. 

## 4. Conclusions

Malaria is still the commonest cause of fever in patients requiring hospitalization after returning from tropical areas [13]. However, malaria is a multisystem disorder which can mimic many diseases and may present with atypical signs and symptoms. 

Hyperglycaemia is a clinical entity not frequently reported in critically sick patients in contrast to hypoglycaemia that is a more common finding and it is postulated to be caused both by the disease process and also secondary to quinine therapy. 

The physicians should focus their attention on this entity because the strong association between hyperglycemia and cerebral malaria and/or other most severe form of malaria with high mortality detail a life-threatening form of severe diseases. 

Moreover, in our patient the progression to severe stages was due to delay in diagnosis of malaria. An increasing awareness of malaria by physicians on early diagnosis and treatment may prevent the progression to severe stages and a prompt intervention in critical situations can reduce the risk of complications or sequelae and could reduce mortality.
